# The enzyme activities of Caf1 and Ccr4 are both required for deadenylation by the human Ccr4–Not nuclease module

**DOI:** 10.1042/BJ20150304

**Published:** 2015-06-19

**Authors:** Maryati Maryati, Blessing Airhihen, G. Sebastiaan Winkler

**Affiliations:** * School of Pharmacy, University of Nottingham, East Drive, University Park, Nottingham NG7 2RD, U.K.

**Keywords:** Ccr4–Not, deadenylase, messenger ribonucleic acid (mRNA) decay, poly(A), post-transcriptional gene regulation, ribonuclease

## Abstract

In eukaryotic cells, the shortening and removal of the poly(A) tail (deadenylation) of cytoplasmic mRNA is a key event in regulated mRNA degradation. A major enzyme involved in deadenylation is the Ccr4–Not deadenylase complex, which can be recruited to its target mRNA by RNA-binding proteins or the miRNA repression complex. In addition to six non-catalytic components, the complex contains two enzymatic subunits with ribonuclease activity: Ccr4 and Caf1 (Pop2). In vertebrates, each deadenylase subunit is encoded by two paralogues: Caf1, which can interact with the anti-proliferative protein BTG2, is encoded by *CNOT7* and *CNOT8*, whereas Ccr4 is encoded by the highly similar genes *CNOT6* and *CNOT6L*. Currently, it is unclear whether the catalytic subunits work co-operatively or whether the nuclease components have unique roles in deadenylation. We therefore developed a method to express and purify a minimal human BTG2–Caf1–Ccr4 nuclease sub-complex from bacterial cells. By using chemical inhibition and well-characterized inactivating amino acid substitutions, we demonstrate that the enzyme activities of Caf1 and Ccr4 are both required for deadenylation *in vitro*. These results indicate that Caf1 and Ccr4 cooperate in mRNA deadenylation and suggest that the enzyme activities of Caf1 and Ccr4 are regulated via allosteric interactions within the nuclease module.

## Abstract (precis)

Enzymatic shortening of mRNA poly(A) tails is important for translation and mRNA degradation. Ccr4–Not, a major eukaryotic deadenylase, contains two catalytic subunits. Using a nuclease module reconstituted with human subunits, we demonstrate that both nuclease subunits are required for deadenylation.

## INTRODUCTION

In eukaryotic cells, virtually all mature cytoplasmic mRNAs are characterized by the presence of a 3′ poly(A) tail. This feature is important for translation and mRNA stability [1,2]. The shortening and removal of the poly(A) tail (deadenylation) is the initial and often rate-limiting step in regulated mRNA decay. Subsequent decapping exposes both ends of the mRNA to exonucleolytic degradation involving the Xrn1 nuclease (5′-3′) and/or the multi-subunit exosome complex (3′-5′ decay) [3–5].

The majority of mRNA deadenylation is carried out by the Pan2–Pan3 and Ccr4–Not enzyme complexes [6–9]. The Ccr4–Not complex contains two catalytic and six accessory subunits [8,10,11]. The ribonuclease activity of the Ccr4 subunit (encoded by the *CNOT6* and *CNOT6L* paralogues in vertebrates) is provided by an endonuclease-exonuclease-phosphatase (EEP) domain [12–15]. Its N-terminal leucine-rich repeat (LRR) domain binds directly to the Caf1 catalytic subunit (encoded by *CNOT7* and *CNOT8* in vertebrates), which is characterized by a RNAseD DEDD domain [15–18]. The nuclease subunits are tethered to the non-catalytic subunits via interactions between Caf1 and the central MIF4G domain of the large subunit, CNOT1 (Not1) [19,20]. This subunit also plays critical roles in the selective recruitment of the Ccr4–Not complex to target mRNAs as exemplified, for instance, by interactions with the RNA-binding proteins tristetraprolin (TTP) and Nanos [21–24]. Moreover, CNOT1 and the non-catalytic RQCD1 (Rcd1/CNOT9) subunit interact with TNRC6 (GW182) thereby facilitating miRNA-mediated mRNA deadenylation and translational repression [25–29]. In addition to selective recruitment to target mRNAs, the Ccr4–Not complex can also be recruited to mRNA via interactions with the conserved N-terminal BTG domains of Tob1 and Tob2 [30–33]. These highly related proteins contain a PAM2 motif facilitating binding to the C-terminal domain of cytoplasmic poly(A)-binding protein [30]. However, other members of the BTG/TOB family of proteins also interact with the Caf1 subunit, including BTG2, but do not possess a PAM2 motif [34–39]. The BTG2 protein is required for the deadenylation of at least several mRNAs [35]. Moreover, its anti-proliferative activity requires the interaction with Caf1, suggesting that the ability of BTG2 to inhibit cell cycle progression is mediated via deadenylation by Ccr4–Not [39].

Currently, it is unclear whether the Ccr4 and Caf1 nuclease subunits have specialized roles or whether they co-operate in mRNA deadenylation. In the yeast *Saccharomyces cerevisiae*, Ccr4 is the main catalytic subunit [12,13]. In agreement with this notion, point mutations that inactivate the catalytic activity of Caf1 complement the phenotype of *Caf1Δ* cells and do not affect deadenylation [40]. However, the enzyme activity of Caf1 contributes to deadenylation in other eukaryotes, including the fission yeast *Schizosaccharomyces pombe* and the filamentous yeast *Aspergillus nidulans* [41,42]. In human cells, there are marked differences in the genome-wide expression profiles of Caf1 and Ccr4-knockdown cells, suggesting that the Caf1 and Ccr4 subunits have unique roles in the regulation of mRNA levels [43,44]. Interestingly, the active sites of Caf1 and Ccr4 are not in close proximity in the X-ray structure of a minimal nuclease module consisting of the budding yeast Not1 MIF4G domain, Caf1 and Ccr4 [19].

To obtain more insight into the mechanism of deadenylation and the relative contributions of the Caf1 and Ccr4 nuclease subunits, we developed a method for the expression and purification of a human BTG2–Caf1–Ccr4 nuclease sub-complex. By using well-characterized single amino acid substitutions that abolish the nuclease activity of Caf1 or Ccr4, we demonstrate that both catalytic subunits are required for deadenylation. This conclusion was corroborated by using small molecules that selectively inhibit Caf1 and do not affect the activity of the catalytic domain of Ccr4.

## MATERIALS AND METHODS

### Plasmids, DNA cloning and site-directed mutagenesis

Plasmids pQE80L (Qiagen) containing codon-optimized cDNAs (Genscript) encoding human Caf1/CNOT7 or Ccr4b/CNOT6LΔLRR (Ccr4b/CNOT6L lacking residues 1–155) were described before [45]. A plasmid containing a codon-optimized cDNA fragment encoding human Ccr4a/CNOT6 lacking the N-terminal LRR domain (amino acids 1–155) was obtained using standard PCR techniques and cloned into the multiple cloning site of pQE80L (Qiagen) using the BamHI and SalI restriction endonucleases. A human BTG2 cDNA containing a BamHI site at the 5′ end and an XhoI site at the 3′ end was amplified using standard techniques and inserted into the BamHI and SalI restriction sites of pQE80L (Qiagen).

Dual expression vectors containing the *CNOT6L* and *CNOT7* cDNAs were generated by first inserting a PCR-generated *CNOT7* cDNA fragment containing a 5′ BamHI and 3′ SalI restriction site into the BglII and XhoI sites of multiple cloning site 2 of vector pACYCDuet-1 (Merck Millipore). Then, a *CNOT6L* cDNA (generated by PCR) was sub-cloned in-frame with the hexahistidine-tag coding sequences into multiple cloning site 1 of the same vector using the BamHI and SalI restriction sites. Alternatively, a *CNOT6L* cDNA fragment containing a 5′ NcoI site was amplified using standard PCR techniques and sub-cloned into the NcoI and SalI sites of multiple cloning site 1 facilitating the expression of untagged Ccr4b/CNOT6L. GST–CNOT6L or GST–CNOT6 fragments were amplified using standard PCR techniques and sub-cloned into the NcoI and NotI sites of multiple cloning site 1. The generation of cDNAs encoding GST–Ccr4b/CNOT6L and GST–Ccr4a/CNOT6 was facilitated by sub-cloning the *CNOT6L* and *CNOT6* cDNAs into the BamHI and SalI sites of vector pGEX4T1 (GE Healthcare Life Sciences).

Site-directed mutagenesis resulting in the amino acid substitutions D40A (Caf1/CNOT7) and E240A (Ccr4a/CNOT6 and Ccr4b/CNOT6L) was carried out using a modified Quikchange procedure (Stratagene). Oligonucleotide sequences used for mutagenesis were designed using the PrimerX tool (http://www.bioinformatics.org/primerx/).

### Protein expression and purification

The human Caf1/CNOT7, Ccr4b/CNOT6LΔLRR and Ccr4a/CNOT6ΔLRR enzymes were expressed and purified from *Escherichia coli* BL21 (DE3) using procedures described before [45]. The trimeric nuclease module was purified following co-expression of His•BTG2, Caf1 and Ccr4 or GST•Ccr4 in *E. coli* strain BL21 (DE3). Cells carrying plasmids pQE80L-BTG2 and pACYCDuet-1/CNOT6L/CNOT7 were grown in lysogeny broth containing 34 μg/ml chloramphenicol and 100 μg/ml ampicillin. Protein expression (4 l culture) was induced by the addition of IPTG (0.2 mM final concentration) for 3 h at 30°C or overnight at room temperature (0.1 mM IPTG, final concentration). Cells were harvested by centrifugation and resuspended in 0.01 volume lysis buffer (20 mM Tris/HCl, pH 7.8, 500 mM NaCl, 10% glycerol, 2 mM 2-mercaptoethanol). Cells were lysed on ice using a Qsonica XL2000 sonicator (40% amplitude) using five 30-s on/30-s off cycles. The crude lysate was cleared by centrifugation using a Sorvall SS-34 rotor spun at 10000 rpm, 4°C for 30 min. Protein complexes were purified from the soluble lysate using Co^2+^-agarose (1 ml bed volume) as described before [45]. Then, peak fractions containing His•BTG2–Caf1–Ccr4 complexes were further purified by gel filtration (Superdex 200 16/60; GE Healthcare Life Sciences) to separate His•BTG2–Caf1 dimeric complexes and trimeric His•BTG2–Caf1–Ccr4 complexes. The column was run in buffer containing 20 mM Tris/HCl (pH 7.8), 150 mM NaCl, 5% (v/v) glycerol and 1 mM 2-mercaptoethanol while collecting 2.5 ml fractions. Alternatively, Pierce GST spin columns (Thermo Scientific) were used as a second affinity step to isolate trimeric His•BTG2–Caf1–GST•Ccr4 complexes following the manufacturer's instructions. Purified proteins were stored in small aliquots at −80°C. Protein concentrations were determined using the Protein Assay Reagent (Bio-Rad).

### SDS/PAGE and immunoblotting

Proteins were analysed by SDS/PAGE (14% gel) followed by staining with Coomassie Blue (SimplyBlue Safestain) or SYPRO Ruby as per the manufacturer's instructions (Life Technologies). For immunoblotting, proteins were transferred to nitrocellulose membranes. Anti-CNOT7, anti-CNOT6L and anti-CNOT6 polyclonal primary antibodies were obtained by immunizing rabbits with peptide-conjugated KLH (Eurogentec). BTG2 was detected using rabbit polyclonal antibody H-50 (Santa Cruz). Horseradish peroxidase-conjugated secondary antibodies (Santa Cruz) were used for detection in combination with an enhanced chemiluminescence detection kit (Pierce). Signals were captured using a Fujifilm LAS-4000 digital imaging system. Image analysis was carried out using ImageJ (http://imagej.nih.gov/ij/).

### Analysis of deadenylase activities

Fluorescence-based analysis of deadenylase activity was carried out as described [45]. Briefly, reaction mixtures (10 μl; 20 mM Tris/HCl, pH 7.9, 50 mM NaCl, 2 mM MgCl_2_, 10% glycerol, 1 mM 2-mercaptoethanol in nuclease-free water) containing 1.0 μM 5′-Flc-labelled RNA substrate and the indicated amount of protein were incubated at 30°C for 60 min. Then, reactions were stopped by the addition of 10 μl of SDS/probe mix containing 1% SDS and a 5-fold molar excess of 3′-labelled DNA probe. The 5′-fluorescein (Flc)-CCU UUC CAA AAA AAA A-3′ RNA substrate oligonucleotide (HPLC purified) and the 5′-TTT TTT TTT GGA AAG G-3′ DNA probe containing a 3′ tetramethylrhodamine (TAMRA) label (HPLC purified) were obtained from Eurogentec. Fluorescence intensity was measured at 25°C using a BioTek Synergy HT plate reader with 96 or 384 U-shaped black multiwell plates. Filter sets used were: 485±20 nm (excitation) and 528±20 nm (detection).

For gel-based analysis of reaction products, 6 μl of RNA loading buffer (95% formamide, 0.025% Bromophenol Blue, 0.025% Xylene Cyanol FF, 0.025% SDS and 5 mM EDTA) was added to a 5 μl of reaction sample and heated for 3 min at 85°C. Part of the RNA mixture (3 μl) was analysed by denaturing PAGE using a 20% acrylamide–bisacrylamide (19:1)/50% (w/v) urea gel (8 cm × 8 cm × 0.1 cm). The gel was run in 0.5× TBE at 200 V using an Xcell mini system (Life Technologies). 5′-Flc-labelled RNA was visualized by epifluorescence using a Fujifilm LAS-4000 system.

### Chemicals

Selective small molecule inhibitors of the Caf1/CNOT7 enzyme NCC-00007277 (*N*-(4-chlorophenyl)-5-[2-[[2-(2-furyl)-1-methyl-ethyl]amino]-2-oxo-ethyl]sulfanyl-1,3,4-thiadiazole-2-carboxamide; ChemDiv), NCC-00001590 (*N*-[6-(isobutylsulfamoyl)-1,3-benzothiazol-2-yl]-2-methyl-benzamide; ChemDiv) and NCC-00039069 (*N*′-[2-[4-[(2-methoxyphenyl)carbamoyl]anilino]-2-oxo-ethyl]-*N*-(2-thienylmethyl)oxamide; Enamine) were as described before [45].

## RESULTS

### Purification of a human BTG2–Caf1–Ccr4b deadenylase sub-complex

**Figure 1:**
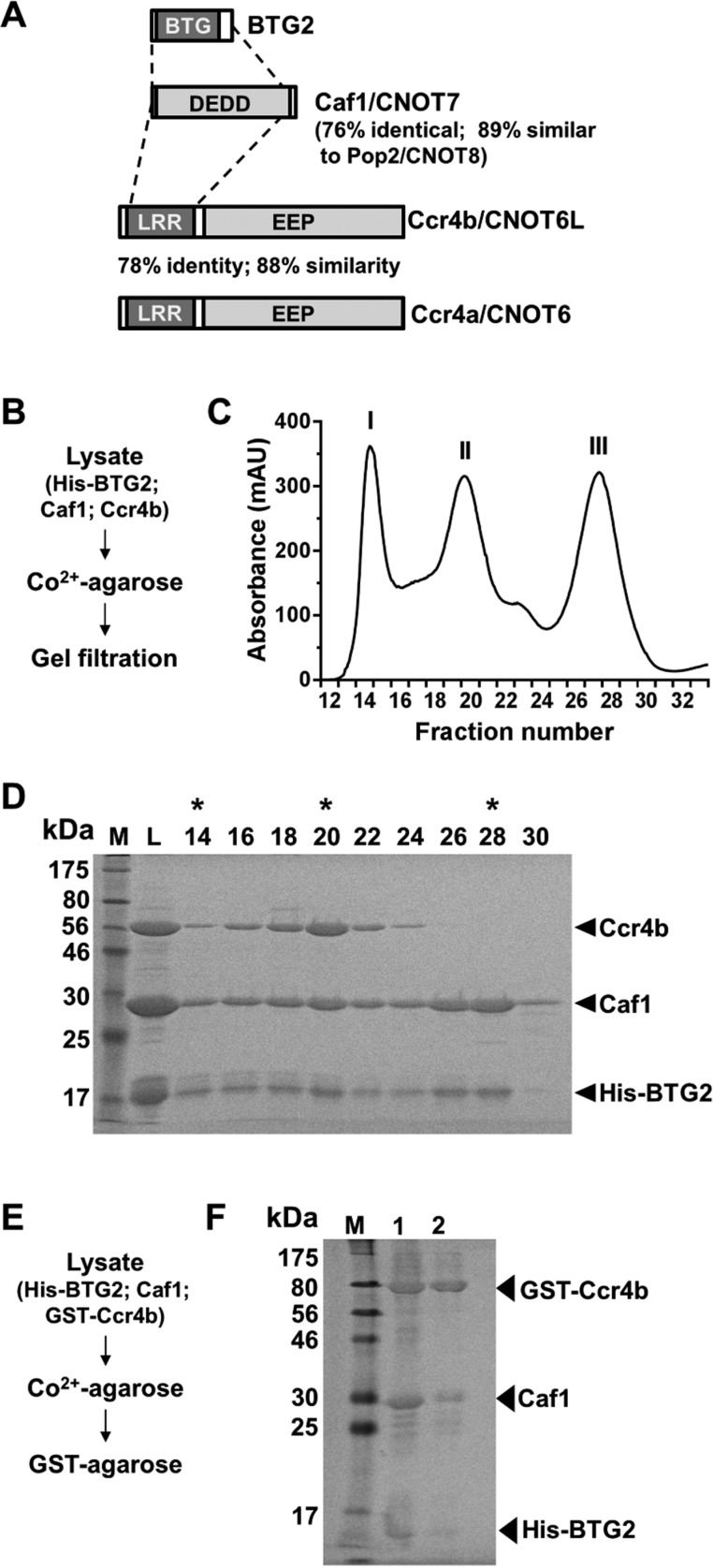
Purification of a human BTG2–Caf1–Ccr4b deadenylase sub-complex (**A**) Diagram of the nuclease subunits Ccr4 and Caf1 and the Caf1-interacting protein BTG2. Shaded in light grey are the DEDD and EEP nuclease domains of Caf1 and Ccr4. The BTG and LRR interaction domains of BTG2 and Ccr4, are indicated in dark grey. (**B**) Purification strategy based on co-expression of His•BTG2, Caf1 and Ccr4b followed by immobilized-metal affinity and size exclusion chromatography. (**C**) Elution profile of the gel filtration step. Peak fractions of the Co^2+^-affinity purified proteins were loaded on to a Superdex 200 16/60 column. Elution fractions (2.5 ml) are indicated on the horizontal axis. (**D**) Analysis of gel filtration elution fractions. Samples were separated by SDS/PAGE (14% gel) and stained with Coomassie Blue. Indicated are the load (L) and elution fractions. Peak fractions containing aggregates (I), trimeric His•BTG2–Caf1–Ccr4b complexes (II) and dimeric His•BTG2–Caf1 complexes (III) are highlighted with asterisks. (**E**) Alternative purification strategy based on co-expression of His•BTG2, Caf1 and GST•Ccr4b followed by subsequent immobilized-metal and glutathione affinity chromatography. (**F**) Purification of His•BTG2–Caf1–GST•Ccr4b by subsequent immobilized-metal (lane 1) and glutathione affinity chromatography (lane 2). Proteins were separated by SDS/PAGE (14% gel) and stained with Coomassie Blue.

To obtain more insight into deadenylation by the Ccr4–Not complex and the relative contributions of the Caf1 and Ccr4 nuclease subunits, we evaluated several strategies for the expression and purification of the nuclease sub-complex containing the Caf1 and Ccr4 catalytic components. Our attempt to reconstitute a Caf1–Ccr4 complex by purifying the isolated components was unsuccessful, because GST fusion proteins of full-length Ccr4 were insoluble in bacterial lysates. Co-expression of His- or GST-tagged Ccr4b (CNOT6L) and Caf1 was also unsuccessful, because we were only partially able to remove a putative chaperone contamination by treatment with ATP and urea. We then co-expressed Caf1, Ccr4b and His-tagged BTG2, whose interaction with Caf1 is well characterized (Figures 1A and 1B) [34–38]. Following consecutive immobilized metal affinity chromatography and gel filtration, trimeric BTG2–Caf1–Ccr4b and dimeric BTG2–Caf1 complexes were obtained (Figures 1C and 1D).

As an alternative purification strategy, we also co-expressed GST•Ccr4b, Caf1 and His-tagged BTG2 (Figure 1E). Following sequential immobilized metal and glutathione affinity chromatography, a highly purified trimeric BTG2–Caf1–Ccr4b complex was obtained (Figure 1F). This two-step procedure is rapid and multiple purifications can be carried out in parallel.

**Figure 2:**
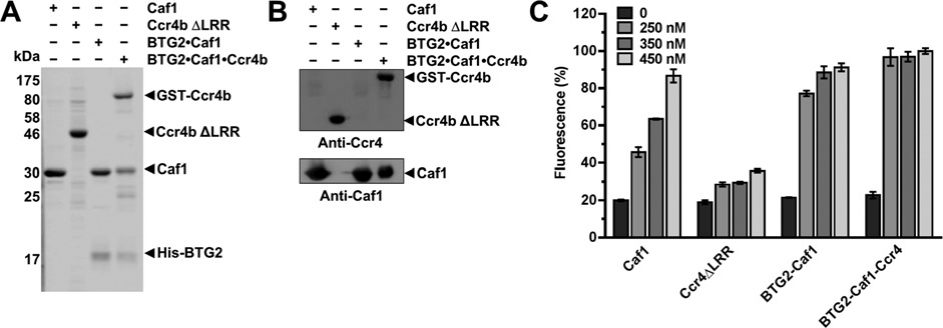
Comparison of deadenylase activities of Caf1, BTG2–Caf1, Ccr4b ΔLRR and the trimeric BTG2–Caf1–Ccr4b complex (**A**) Analysis of purified deadenylase subunits and protein complexes. Purified Caf1, Ccr4b ΔLRR, dimeric BTG2–Caf1 and trimeric BTG2–Caf1–Ccr4b complexes (2.0 μg) were separated by SDS/PAGE (14% gel) and stained with SYPRO Ruby. (**B**) Immunoblot analysis of purified deadenylase subunits and protein complexes. Purified Caf1, Ccr4b ΔLRR, BTG2–Caf1 and BTG2–Caf1–Ccr4b proteins were separated by SDS/PAGE (14% gel) and transferred to nitrocellulose membranes. Proteins were detected by immunoblotting using antibodies recognizing Ccr4b (top) and Caf1 (bottom). (**C**) Comparison of the deadenylase activity of purified Caf1, Ccr4b ΔLRR, BTG2–Caf1 and BTG2–Caf1–Ccr4b. The indicated amount of protein was incubated at 30°C for 60 min. Error bars indicate the standard error of the mean (S.E.M.) (*n*=3).

### Comparison of the deadenylase activities of Caf1, BTG2–Caf1, Ccr4b ΔLRR and the trimeric BTG2–Caf1–Ccr4b complex

As a first step to evaluate the contributions of the Caf1 and Ccr4 subunits to the ribonuclease activity of the Ccr4–Not complex, we compared the deadenylase activity of the dimeric BTG2–Caf1 complex, the trimeric BTG2–Caf1–Ccr4 module and those of Caf1 and Ccr4b lacking the LRR domain (Ccr4b ΔLRR). Analysis of the purified proteins by SDS/PAGE indicated that they were of comparable purity, although the concentration of the trimeric BTG2–Caf1–Ccr4 module was somewhat overestimated as compared with the other purified proteins (Figure 2A). This was confirmed by immunoblot analysis (Figure 2B). We then determined the activity of the protein samples using a recently developed fluorescence-based deadenylase assay [45]. The method is based on the incubation of enzyme and a 5′ Flc-labelled RNA substrate, followed by the addition of a complementary DNA probe containing a 3′-carboxy TAMRA label. In the absence of deadenylase activity, addition and subsequent annealing of the probe will result in quenching of Flc fluorescence, due to the close proximity of the TAMRA moiety. By contrast, efficient annealing of the DNA probe is prevented when the substrate is degraded, thus allowing detection of Flc-mediated fluorescence [45]. Using this assay, we found that Caf1 and Ccr4b ΔLRR both displayed deadenylase activity, as expected. However, we found that Caf1 displays significantly higher activity as compared with the catalytic domain of Ccr4b (Figure 2C). Unexpectedly, the dimeric BTG2–Caf1 complex displayed increased activity as compared with monomeric Caf1. In addition, the activity of the trimeric BTG2–Caf1–Ccr4b complex was more active than any of the other purified components, despite the fact that its concentration was somewhat lower (Figure 2C). Taken together, we conclude that the enzyme activities of Caf1 and Ccr4 both contribute to deadenylation within the context of the nuclease sub-complex.

### The catalytic activities of Caf1 and Ccr4b are both required for deadenylation by the BTG2–Caf1–Ccr4b nuclease module

**Figure 3:**
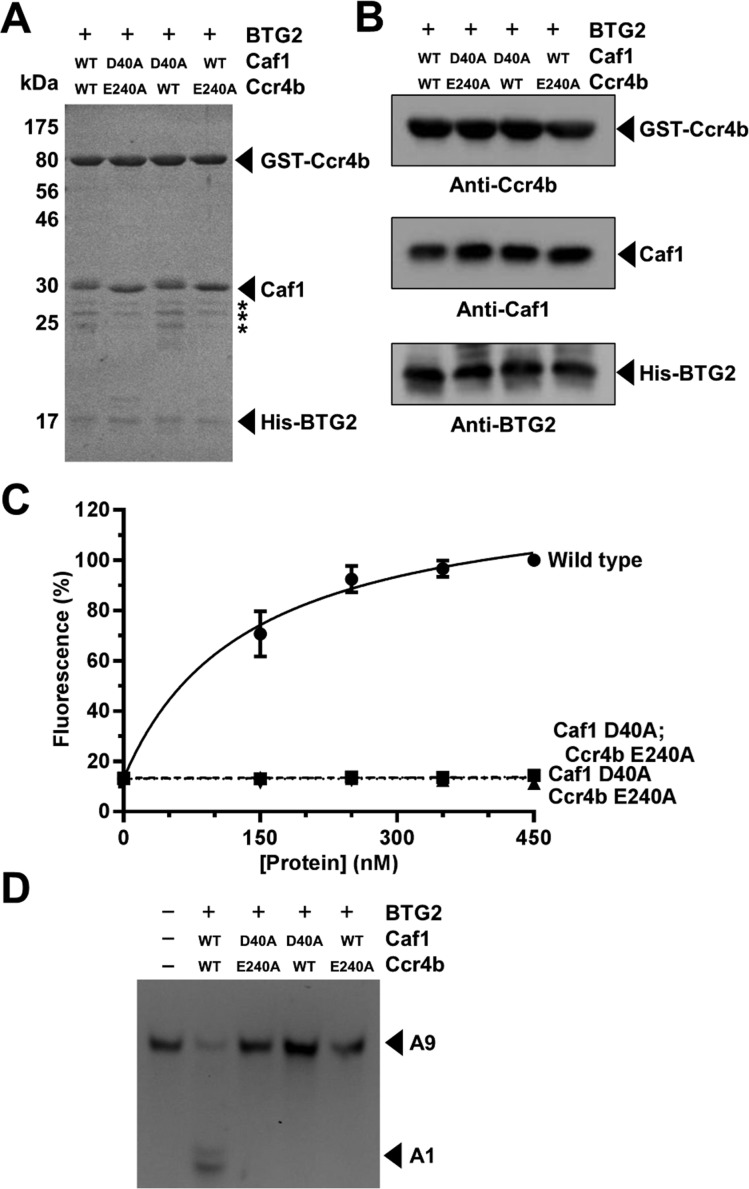
The catalytic activities of Caf1 and Ccr4b are both required for deadenylation by the BTG2–Caf1–Ccr4b nuclease module (**A**) Purification of BTG2–Caf1–Ccr4b nuclease modules containing catalytically inactive deadenylase subunits. Amino acid substitutions abolishing the nuclease activity of Caf1 (D40A) or Ccr4b (E240A) are indicated. Proteins (2.0 μg) were separated by SDS/PAGE and stained with Coomassie Blue. Minor contaminants are indicated by asterisks. (**B**) Immunoblot analysis of purified His•BTG2–Caf1–GST•Ccr4b nuclease sub-complexes. Proteins were detected using antibodies recognizing Ccr4b, Caf1 and BTG2. (**C**) Comparison of the deadenylase activity of purified BTG2–Caf1–Ccr4b complexes. Amino acid substitutions inactivating the nuclease activity of Caf1 (D40A) and Ccr4b (E240A) are indicated. The indicated amount of protein was incubated at 30°C for 60 min. Error bars indicate the S.E.M. (*n*=3). (**D**) Product analysis by PAGE. A fluorescent RNA oligonucleotide containing nine terminal adenosine residues (A9) was used as a substrate for purified BTG2–Caf1–Ccr4b complexes. Proteins (450 nM) were incubated with RNA substrate (1.0 μM) at 30°C for 60 min. Amino acid substitutions abolishing the nuclease activities of Caf1 (D40A) and Ccr4b (E240A) and the positions of the intact RNA substrate (A9) and the degradation product (A1) are indicated.

To establish the relative contributions of the Caf1 and Ccr4b subunits to deadenylation by the trimeric nuclease module, we used site-directed mutagenesis to introduce the amino acid substitutions D40A and/or E240A, which abolish the catalytic activity of Caf1 and Ccr4b respectively. We then purified BTG2–Caf1–Ccr4b complexes containing either wild-type or inactive Caf1 and/or Ccr4b using subsequent immobilized metal and glutathione affinity chromatography (Figure 3A). Analysis of the purified proteins by SDS/PAGE indicated that the protein complexes were of comparable purity and concentration (Figure 3A), which was confirmed by immunoblot analysis (Figure 3B). Surprisingly, the deadenylase activity of complexes containing either inactive Caf1 (D40A) or inactive Ccr4b (E240A) was undetectable and indistinguishable from the background signal observed with complexes in which both Caf1 and Ccr4 were inactive (Figure 3C). Product analysis by denaturing PAGE indicated that these observations were not due to artefacts of the fluorescence assay (Figure 3D). Together, these findings indicate that both Caf1 and Ccr4b are required for deadenylation by a trimeric BTG2–Caf1–Ccr4b nuclease sub-complex.

### Selective inhibitors of Caf1 inhibit the deadenylase activity of a BTG2–Caf1–Ccr4b trimeric nuclease module

**Figure 4:**
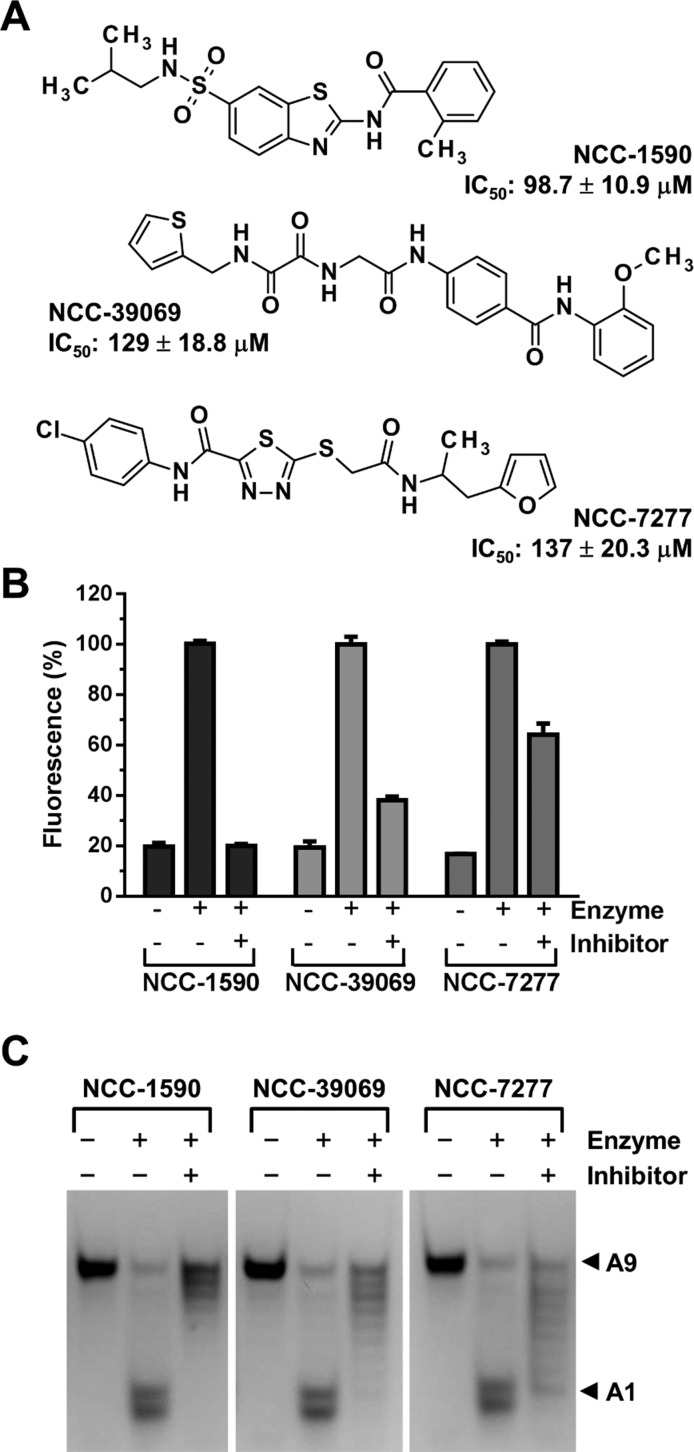
Selective inhibitors of Caf1 inhibit the deadenylase activity of the BTG2-Caf1-Ccr4b trimeric nuclease module (**A**) Structure and IC_50_ values of selective inhibitors of Caf1. IC_50_ values were determined using isolated Caf1 [45]. (**B**) The deadenylase activity of the trimeric BTG2–Caf1–Ccr4b complex was assessed in the presence of the indicated compounds (300 μM final concentration). Protein complexes were pre-incubated with the indicated compounds at room temperature for 15 min. After addition of Flc-labelled substrate RNA, reaction mixtures were incubated at 30°C for 60 min. Fluorescence was measured after addition of a mixture containing SDS (0.5% final concentration) and a 5-fold molar excess of TAMRA-labelled probe. Error bars indicate the S.E.M. (*n*=3). (**C**) Product analysis using PAGE. A fluorescent RNA oligonucleotide containing nine terminal adenosine residues (A9) was used as a substrate for purified BTG2–Caf1–Ccr4b complexes (450 nM). The positions of the intact RNA substrate (A9) and the degradation product (A1) are indicated.

To explore the requirement of Caf1 in deadenylation by a trimeric BTG2–Caf1–Ccr4b complex in more detail, we used selective Caf1 inhibitors [45]. Using a panel of Caf1 inhibitors identified before, we selected three compounds that are unable to inhibit the activity of the Ccr4 ΔLRR enzyme [45]. Because of their potency compared with isolated Caf1 (IC_50_ values in the range of 100–140 μM; Figure 4A), we used a single concentration of 300 μM for each compound. As shown (Figures 4A and 4B), the most potent compound (NCC-1590; IC_50_=98.7±10.9 μM) abolished the activity of the trimeric complex. Lower potency compounds NCC-39069 (IC_50_=129±18.8 μM) and NCC-7277 (IC_50_=137±20.3 μM) partially inhibited the activity of the BTG2–Caf1–Ccr4b complex. These results indicate that Caf1 makes a major contribution to the deadenylase activity of the BTG2–Caf1–Ccr4b complex and are consistent with the conclusion that Caf1 is required for the activity of the trimeric complex.

### Deadenylation by the BTG2–Caf1–Ccr4a complex

**Figure 5:**
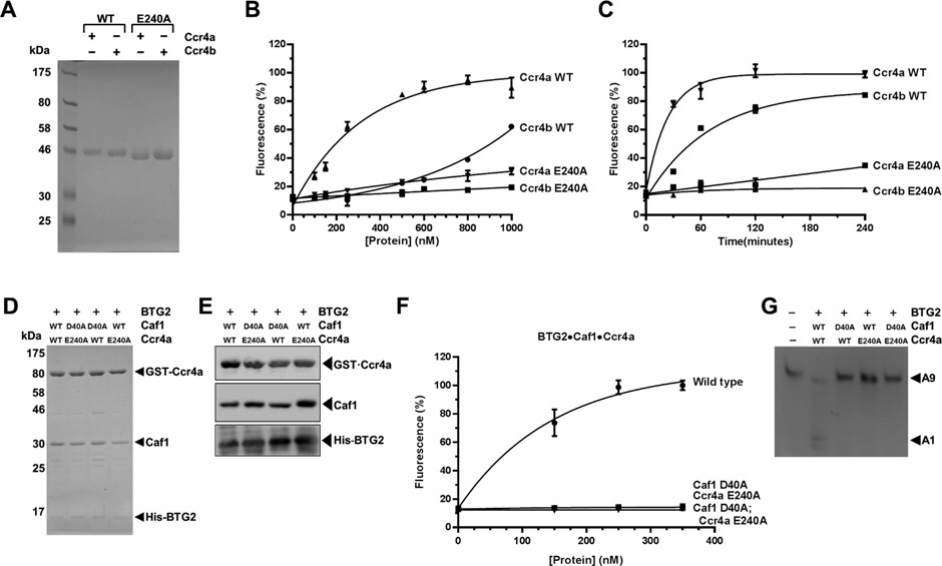
Deadenylation by the BTG2–Caf1–Ccr4a complex (**A**) Analysis of purified wild-type and inactive Ccr4a ΔLRR and Ccr4b ΔLRR. The inactivating amino acid substitution E240A is indicated. Proteins were expressed in *E. coli* and purified by immobilized metal affinity chromatography. Proteins (2.0 μg) were analysed by SDS/PAGE (14% gel) and stained with Coomassie Blue. (**B**) Comparison of the deadenylase activities of Ccr4a ΔLRR and Ccr4b ΔLRR. Increasing amounts of the indicated proteins were incubated at 30°C for 60 min. Error bars indicate the S.E.M. (*n*=3). (**C**) Time-course comparison of Ccr4a ΔLRR and Ccr4b ΔLRR. The indicated proteins (1.0 μM) were incubated at 30°C for the indicated amount of time. Error bars indicate the S.E.M. (*n*=3). (**D**) Purification of BTG2–Caf1–Ccr4a nuclease modules lacking catalytically active deadenylase subunits. Amino acid substitutions abolishing the nuclease activity of Caf1 (D40A) and Ccr4a (E240A) are indicated. Proteins (2.0 μg) were separated by SDS/PAGE (14% gel) and stained with Coomassie Blue. (**E**) Immunoblot analysis of purified BTG2–Caf1–Ccr4a nuclease sub-complexes. Proteins were detected using antibodies recognizing Ccr4a, Caf1 and BTG2. (**F**) Comparison of the deadenylase activity of purified BTG2–Caf1–Ccr4a complexes. Amino acid substitutions disabling the nuclease activity of Caf1 (D40A) and Ccr4a (E240A) are indicated. Proteins were incubated at 30°C for 60 min. Error bars indicate the S.E.M. (*n*=3). (**G**) Product analysis by PAGE. A fluorescent RNA oligonucleotide containing nine adenosines (A9) was used as a substrate for purified BTG2–Caf1–Ccr4a complexes. Proteins (350 nM) were incubated with RNA substrate (1.0 μM) at 30°C for 60 min. Amino acid substitutions inactivating the nuclease activity of Caf1 (D40A) and Ccr4a (E240A) and the positions of the intact RNA substrate (A9) and the degradation product (A1) are indicated.

To confirm the requirement of the nuclease subunits further, we next focused on the Ccr4a (CNOT6) subunit, which is highly related to Ccr4b (78% identity, 88% similarity). We expressed and purified Ccr4a lacking the LRR domain (Ccr4a ΔLRR) as well as a catalytically inactive mutant (Figure 5A). The deadenylase activity of Ccr4a ΔLRR was readily detectable and appeared significantly increased as compared with the activity of Ccr4b ΔLRR (Figures 5B and 5C). We then purified BTG2–Caf1–Ccr4a complexes containing either wild-type or inactive Caf1 and/or Ccr4 using subsequent immobilized metal and glutathione affinity chromatography (Figure 5D). Again, analysis of the purified proteins by SDS/PAGE indicated that the protein complexes were of comparable purity and concentration (Figure 5D), which was confirmed by immunoblot analysis (Figure 5E). In addition, as was the case when characterizing the BTG2–Caf1–Ccr4b nuclease modules, the deadenylase activity of BTG2–Caf1–Ccr4a complexes containing either inactive Caf1 (D40A) or inactive Ccr4a (E240A) was undetectable and indistinguishable from the background signal observed with complexes in which both Caf1 and Ccr4a were inactive (Figure 5F). Again, product analysis by denaturing PAGE indicated that these observations were not due to artefacts of the fluorescence assay (Figure 5G).

Taken together, the results demonstrate that (1) a complex containing Caf1 and Ccr4 is more active than its isolated components; and (2) both Caf1 and Ccr4 are required for deadenylation by a trimeric BTG2–Caf1–Ccr4 nuclease sub-complex *in vitro*. In addition, in agreement with a positive role in deadenylation [35], BTG2 does not appear to inhibit the enzyme activity of Caf1.

## DISCUSSION

The Ccr4–Not complex is a major deadenylase enzyme involved in the shortening and removal of the poly(A) tail of cytoplasmic mRNA. It is equipped with two catalytic subunits containing ribonuclease activity that display selectivity for poly(A) residues. However, it has been unclear whether the catalytic nuclease subunits co-operate in deadenylation or whether they have unique roles. Here, we provide evidence that the ribonuclease activities of Caf1 and Ccr4 are both required for deadenylation. The findings are based on a newly developed strategy for the expression and purification of a trimeric nuclease complex composed of the human anti-proliferative BTG2 protein, Caf1 and Ccr4. This allowed the analysis of purified complexes containing one inactive nuclease subunit (either Caf1 or Ccr4) or two inactive subunits (both Caf1 and Ccr4). Three independent approaches indicate that the enzyme activities of both subunits are required: (i) the analysis of BTG2–Caf1–Ccr4b complexes; (ii) the use of selective inhibitors of Caf1 [45], which are able to completely inhibit the activity of trimeric BTG2–Caf1–Ccr4b; and (iii) the analysis of BTG2–Caf1–Ccr4a nuclease modules. It should be noted that even at the highest enzyme concentrations, multiple rounds of catalysis (>10) are required for the complete degradation of the substrate.

The conclusion that the enzyme activities of both Caf1 and Ccr4 are required is surprising, because several results indicated that the nuclease subunits have unique roles. First, the catalytic activity of Caf1 is dispensable in *Saccharomyces cerevisiae*, indicating that the enzyme activity of Ccr4 is sufficient for deadenylation [12,13,40]. In addition, knockdown of the Caf1 paralogues in human cells differentially affects gene expression as compared with knockdown of the Ccr4 paralogues [43,44]. Also, the isolated, monomeric versions of Caf1 protein and the purified EEP domain of Ccr4 are active ribonuclease enzymes. Finally, the structural analysis of a minimal nuclease module composed of the yeast MIF4G domain of Not1, Caf1 and Ccr4 indicated that the active sites of Caf1 and Ccr4 are not in close proximity [19]. Although we only investigated the role of a nuclease sub-complex, we believe that it is likely that both enzyme activities are also required in the context of the complete Ccr4–Not complex, although we cannot exclude that the accessory subunits of the Ccr4–Not complex modulate the activity of the nuclease module. Regardless, the results reported in the present study reveal an unexpected property of the nuclease sub-complex.

Interestingly, Petit et al. [20] found that the catalytic pocket of Caf1 is occluded by its C-terminus (residues G^274^-E^280^) in the X-ray structure of Caf1 in complex with the MIF4G domain of CNOT1 [20]. Although the authors indicated that this simply may be due to the conditions required for crystal packing, they also raised the possibility that this was a potential mechanism for regulation of the deadenylase activity of Caf1.

Analysis of the activities of monomeric subunits indicated that the deadenylase activity associated with the EEP domain of Ccr4a is more active as compared with the Ccr4b nuclease domain, despite their high overall similarity. In addition, we noticed that the BTG2–Caf1 dimeric complex displays a higher activity as compared with the isolated Caf1 protein, whereas the trimeric BTG2–Caf1–Ccr4 complexes display even higher activity. This was surprising, as it was reported that BTG2 is able to inhibit the deadenylase activity of Caf1 [38]. However, our finding is in agreement with a role for BTG2 as a positive regulator of mRNA deadenylation as well as with the observation that the BTG domain of Tob1 is unable to inhibit the activity of Caf1 [33,35].

Taken together, our data support a model in which the ribonuclease subunits of the Ccr4–Not complex co-operate in deadenylation. We speculate that alternate action of Caf1 and Ccr4 is required. The findings that a complex containing Caf1 and Ccr4 is more active than its isolated components as well as the observations that the enzyme activities of both Caf1 and Ccr4 are required for deadenylation by a BTG2–Caf1–Ccr4 complex suggest a model wherein the catalytic activities of Caf1 and Ccr4 are regulated via allosteric interactions within the nuclease module.

## Abbreviations

EEP: endonuclease-exonuclease-phosphatase
Flc: fluorescein
LRR: leucine-rich repeat
S.E.M.: standard error of the mean
TAMRA: tetramethylrhodamine
